# A Comprehensive Review on the Thermal Stability Assessment of Polymers and Composites for Aeronautics and Space Applications

**DOI:** 10.3390/polym15183786

**Published:** 2023-09-16

**Authors:** Giuseppina Barra, Liberata Guadagno, Marialuigia Raimondo, Maria Gabriella Santonicola, Elisa Toto, Stefano Vecchio Ciprioti

**Affiliations:** 1 Department of Industrial Engineering, University of Salerno, Via Giovanni Paolo II, 132, 84084 Fisciano, Italy; gbarra@unisa.it (G.B.); lguadagno@unisa.it (L.G.); 2 Department of Chemical Engineering Materials Environment, Sapienza University of Rome, Via del Castro Laurenziano 7, 00161 Rome, Italy; elisa.toto@uniroma1.it; 3 Department of Basic and Applied Science for Engineering, Sapienza University of Rome, Via del Castro Laurenziano 7, 00161 Rome, Italy

**Keywords:** thermal stability, polyimides, carbon fillers, epoxy composites

## Abstract

This review article provides an exhaustive survey on experimental investigations regarding the thermal stability assessment of polymers and polymer-based composites intended for applications in the aeronautical and space fields. This review aims to: (1) come up with a systematic and critical overview of the state-of-the-art knowledge and research on the thermal stability of various polymers and composites, such as polyimides, epoxy composites, and carbon-filled composites; (2) identify the key factors, mechanisms, methods, and challenges that affect the thermal stability of polymers and composites, such as the temperature, radiation, oxygen, and degradation; (3) highlight the current and potential applications, benefits, limitations, and opportunities of polymers and composites with high thermal stability, such as thermal control, structural reinforcement, protection, and energy conversion; (4) give a glimpse of future research directions by providing indications for improving the thermal stability of polymers and composites, such as novel materials, hybrid composites, smart materials, and advanced processing methods. In this context, thermal analysis plays a crucial role in the development of polyimide-based materials for the radiation shielding of space solar cells or spacecraft components. The main strategies that have been explored to improve the processability, optical transparency, and radiation resistance of polyimide-based materials without compromising their thermal stability are highlighted. The combination of different types of polyimides, such as linear and hyperbranched, as well as the incorporation of bulky pendant groups, are reported as routes for improving the mechanical behavior and optical transparency while retaining the thermal stability and radiation shielding properties. Furthermore, the thermal stability of polymer/carbon nanocomposites is discussed with particular reference to the role of the filler in radiation monitoring systems and electromagnetic interference shielding in the space environment. Finally, the thermal stability of epoxy-based composites and how it is influenced by the type and content of epoxy resin, curing agent, degree of cross-linking, and the addition of fillers or modifiers are critically reviewed. Some studies have reported that incorporating mesoporous silica micro-filler or microencapsulated phase change materials (MPCM) into epoxy resin can enhance its thermal stability and mechanical properties. The mesoporous silica composite exhibited the highest glass transition temperature and activation energy for thermal degradation among all the epoxy-silica nano/micro-composites. Indeed, an average activation energy value of 148.86 kJ/mol was recorded for the thermal degradation of unfilled epoxy resin. The maximum activation energy range was instead recorded for composites loaded with mesoporous microsilica. The EMC-5p50 sample showed the highest mean value of 217.6 kJ/mol. This remarkable enhancement was ascribed to the polymer invading the silica pores and forging formidable interfacial bonds.

## 1. Introduction

In recent times, more attention has been paid to the thermal stability assessment of different classes of materials (glasses, organic-inorganic hybrids, ceramics, fibers, polymers and composites, etc. [1,2,3,4,5,6,7,8,9]). The demand for the enhanced thermal stability of some kinds of materials is mainly reasonably explained by the need to use them under more severe and critical conditions without losing their main properties [10,11,12,13,14].

In the last few years, much effort has been made in the selection and development of the most effective materials with improved mechanical properties for the aerospace industry [15,16].

Polymers and polymer-based composites seem to be among the most promising materials for applications in the aeronautical and space fields, where the demand for enhanced thermally stable polymers or composites that preserve their useful properties at high temperatures has been extensively increased [17,18,19,20].

As far as space applications are concerned, polymer-based materials must retain their properties in adverse environmental conditions, characterized by a combination of temperature variability and high levels of ionizing radiation under vacuum pressure. In this context, thermal stability is an important property of epoxy resins that affects their processing and application performance.

Thermal analysis techniques, with particular reference to thermogravimetry (TG) and differential thermal analysis (DTA), or differential scanning calorimetry (DSC), are used to assess a thermal stability scale of related materials undergoing decomposition with similar reaction mechanisms [1,3,5]. This result has been mainly fulfilled on the basis of the onset decomposition temperature extrapolated from TG curves [5,21,22]. These techniques are also particularly interesting for studying the thermal behavior of different kinds of materials (i.e., ionic liquids, glasses, plastic waste [23,24,25,26,27,28]), aiming to identify the appropriate temperature ranges under defined atmosphere conditions where some processes occur, which may convert pristine precursors to more active and effective novel materials with promising and interesting properties. Other research methods, such as modern numerical methods based on the finite element method, carried out on composite structures have been developed and successfully employed. In this regard, very captivating papers have presented the results of advanced numerical analyses of layered polymer composites [29,30]. More specifically, the paper [29] presents the results of advanced numerical analyses of layered carbon fiber reinforced polymer (CFRP) composite plates with variable hole diameters under tensile loading. The paper aims to investigate the effect of the hole diameter on the stress distribution, strain distribution, damage initiation, damage propagation, and failure modes of the composite plates. The paper also compares the results of different numerical methods, such as the finite element method (FEM), extended finite element method (XFEM), and cohesive zone method (CZM).

Some of the main results of the article are:

- The FEM, XFEM, and CZM models can accurately capture the nonlinear behavior and progressive damage of the composite plates with different hole diameters.
- The hole diameter has a significant influence on the stress concentration, strain concentration, damage initiation, damage propagation, and failure modes of the composite plates.
- The smaller the hole diameter, the higher the stress concentration and strain concentration around the hole edge, which leads to earlier damage initiation and faster damage propagation.
- The larger the hole diameter, the lower the stress concentration and strain concentration around the hole edge, which leads to delayed damage initiation and slower damage propagation.
- The failure modes of the composite plates vary with the hole diameter, such as matrix cracking, fiber breakage, fiber-matrix debonding, delamination, net-section failure, and pull-out failure.

The paper [30] presents the results of advanced numerical analyses of layered polymer composites based on the finite element method (FEM) and the cohesive zone method (CZM). The paper aims to investigate the stability and failure behavior of compressed composite plates with different geometries, boundary conditions, and material properties. The paper also compares the numerical results with the experimental results obtained from compression tests.

Some of the main results of the paper are:

- The FEM and CZM models can accurately simulate the buckling and post-buckling behavior of compressed composite plates, as well as the damage initiation and propagation in the form of matrix cracking, fiber-matrix debonding, and delamination.
- The stability and failure behavior of compressed composite plates are influenced by several factors, such as the plate aspect ratio, plate thickness, plate curvature, loading type, support type, fiber orientation, and ply stacking sequence.
- The plate aspect ratio has a significant effect on the buckling load and mode, as well as the post-buckling response and failure mode. The higher the aspect ratio, the lower the buckling load and the higher the post-buckling deflection. The failure mode also changes from global buckling to local buckling as the aspect ratio increases.
- The plate thickness has a significant effect on the buckling load and mode, as well as the post-buckling response and failure mode. The thicker the plate, the higher the buckling load and the lower the post-buckling deflection. The failure mode also changes from global buckling to local buckling as the plate thickness increases.
- The plate curvature has a significant effect on the buckling load and mode, as well as the post-buckling response and failure mode. The curved plate has a higher buckling load and a lower post-buckling deflection than the flat plate. The failure mode also changes from global buckling to local buckling as the plate curvature increases.
- The loading type has a significant effect on the buckling load and mode, as well as the post-buckling response and failure mode. The uniformly distributed load has a lower buckling load and a higher post-buckling deflection than the concentrated load. The failure mode also changes from global buckling to local buckling as the loading type changes from uniform to concentrated.
- The support type has a significant effect on the buckling load and mode, as well as the post-buckling response and failure mode. The simply supported plate has a lower buckling load and a higher post-buckling deflection than the clamped plate. The failure mode also changes from global buckling to local buckling as the support type changes from simple to clamped.
- The fiber orientation has a significant effect on the buckling load and mode, as well as the post-buckling response and failure mode. The angle-ply plate has a lower buckling load and a higher post-buckling deflection than the cross-ply plate. The failure mode also changes from global buckling to local buckling as the fiber orientation changes from cross-ply to angle-ply.
- The ply stacking sequence has a significant effect on the buckling load and mode, as well as the post-buckling response and failure mode. The symmetric plate has a higher buckling load and a lower post-buckling deflection than the asymmetric plate. The failure mode also changes from global buckling to local buckling as the ply stacking sequence changes from symmetric to asymmetric.

The main aim of this review paper is to provide an updated and exhaustive survey on the assessment of a thermal stability scale of materials related to polymers and composites used in the field of aeronautics and space applications. An attempt was also made to establish a correlation between the thermal stability and chemical structure of the polymer-based materials. The content of this review has been subdivided into the following three different sections/chapters, entitled: thermal stability of polyimide-based materials for space applications; thermal stability of carbon-filled polymer composites for space applications; thermal stability of epoxy composites tailored for aeronautical and aerospace applications. These sections are followed by a conclusion, which summarizes all of the relevant achievements and shows remarkable future perspectives.

## 2. Thermal Stability of Polyimide-Based Materials for Space Applications

Polyimides (PIs) are a class of high-performing polymers, exhibiting high-temperature resistance, low-temperature tolerance, chemical and radiation durability, and good mechanical and dielectric properties [31,32]. Due to these properties, PIs are often employed in space-related applications. At present, one of the main objectives in the research and development of PIs is to improve their processability and optical transparency without sacrificing their thermal stability. Traditional aromatic PIs show the main disadvantage of deep coloration, which can limit their use for space applications that require optical transparency and lower solar absorption [14]. The deep color of PIs is generally related to the presence of intramolecular and intermolecular charge transfer complexes (CTC), which can be removed by incorporating aliphatic/alicyclic moieties or asymmetric non-coplanar structures, and fluorine elements in the PI main chain [33,34]. It was demonstrated that the introduction of such bulky pendant substituents can improve the processability and optical transparency of PIs without significantly affecting their thermal stability [14].

### 2.1. High Performing Polyimides from Novel Precursors

Wu et al. investigated the properties of PIs synthesized with the incorporation of bulky cyclohexyl and ortho-substituted tertiary butyl groups into the backbone [35]. They produced a novel diamine (CHMBTBA) containing bulky cyclohexyl and ortho-substituted tertiary butyl groups, and prepared different PIs starting from the one-pot polycondensation of CHMBTBA using several aromatic dianhydrides. These polymers were used to fabricate films, and their thermal properties were investigated through DSC, a dynamic mechanical analyzer (DMA), and TG. The DSC and DMA measurements showed values of the glass transition temperature (*T*_g_) in the range of 310–394 °C, whereas the TG results unveiled high thermal stability for all of the PI films, which maintained their weight within 5% up to 480 °C. Therefore, this work demonstrated the high thermal stability of optically transparent PI films obtained by incorporating cyclohexyl and ortho-substituted tertiary butyl groups into the backbone.

**Figure 1:**
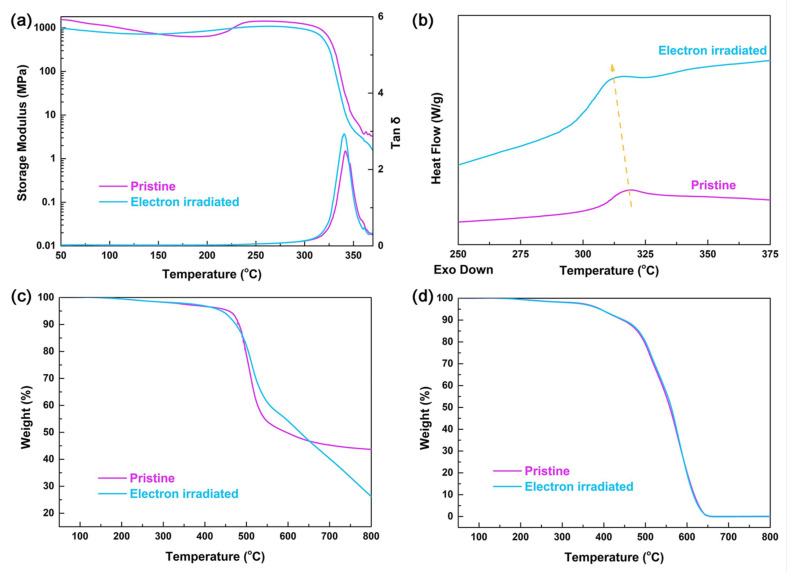
Results from (**a**) DMA, (**b**) DSC, and (**c**,**d**) TG measurements performed in nitrogen atmosphere and air, respectively, on pristine and electron irradiated polyimide films. Reproduced with permission from Ref. [36].

In subsequent work, the properties of these PIs were investigated before and after electron irradiation in order to test their suitability as possible encapsulating materials for space solar cells [36]. The thermal properties of pristine and electron-irradiated PI films were analyzed through DMA, DSC, and TG. The results from the DMA (Figure 1a) showed a decrease in the modelling modulus at two different temperatures, which can be identified as the α relaxation (at the higher temperature) and β relaxation (at the lower temperature) of the polyimide chains. In particular, the α relaxation occurs at the glass transition, whereas the β relaxation corresponds to the rotation or oscillation of the pendant groups or end groups [36,37]. The irradiated polyimides exhibited a lower β relaxation peak temperature (139 °C), which can be ascribed to the scission of the molecular chains due to the energetic electron interaction. The glass transition temperature showed a slight variation, decreasing from 342 to 340 °C (Figure 1a) after irradiation. This behavior was confirmed through DSC analysis, in which a variation from 317 to 313 °C was observed (Figure 1b). The TG measurements performed under nitrogen atmosphere (Figure 1c) unveiled a decrease in the decomposition temperature after electron exposure, from 458 to 442 °C, with the residual mass at 800 °C decreasing from 43 wt% to 26 wt%. In the presence of air, the TG measurements unveiled the same behavior for pristine and non-irradiated films (Figure 1d), indicating that electron irradiation had a negligible effect on the thermal stability of the PIs [35,37].

**Figure 2:**
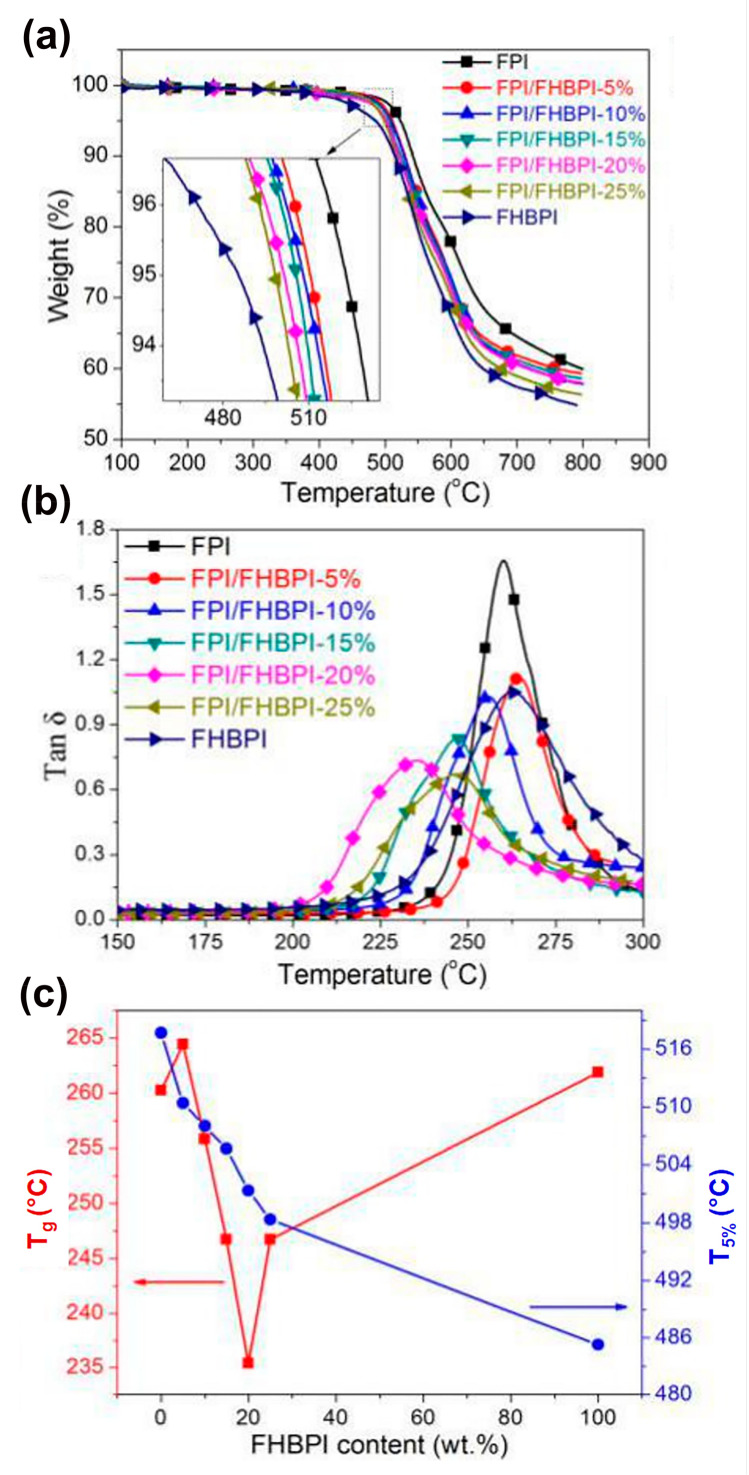
Thermal analysis of FPI, FPI/FHBPI and FHBPI polyimide films performed by (**a**) TG under nitrogen atmosphere and (**b**) DMA; (**c**) variation of *T_g_* and 5% decomposition temperature (*T*_5%_) of the polyimide films as a function of the FHBPI content. Adapted with permission from Ref. [40].

In the research of high-performing polyimides, many works have focused on hyperbranched polyimides (HBPIs), which are characterized by a spherical molecular structure with reactive terminal functional groups [37,38,39,40]. These polymers show appreciable properties, such as good processability, without a significant loss in thermal stability with respect to linear polyimides. Nevertheless, the presence of many benzene rings leads to the formation of CTCs, making these polyimides not optically transparent but UV-shielding. Li et al. successfully integrated fluorinated linear polyimides (FPIs) and fluorinated hyperbranched polyimides (FHBPIs) to improve the mechanical behavior and optical transparency of the resulting polymers, without losses in the thermal stability and UV-shielding properties [14]. Several polyimide films were prepared by varying the FHBPI content. The thermal behavior of the resulting samples was evaluated through DMA and TG, and the results are shown in Figure 2.

Figure 2a shows that none of the samples experienced mass loss before 400 °C, with the pure FPI films exhibiting the best thermal stability. As the amount of FHBPI increased, a loss in the thermal stability was observed. The FHBPI films exhibited lower thermal properties, and this can be ascribed to the hyperbranched structure of FHBPI, with more irregular terminal anhydride than FPI. The *T*_5%_ (Figure 2c) and *T*_10%_ of the samples with a FHBPI content of 25 wt% are 498.3 and 516.2 °C, respectively, indicating that such films are suitable for use as heat resistant materials. Figure 2b shows the presence of only one peak in the tanδ curve of the FPI/FHBPI samples, indicating excellent compatibility between FPI and FHBPI. For these films, the variation of the *T*_g_ as a function of the FHBPI content is shown in Figure 2c. This variation can be ascribed to the restriction in the movements of the molecular segments. Nevertheless, the *T*_g_ of the FPI/FHBPI samples can be considered high enough to meet the basic requirements for use as heat resistant materials. Based on these results, the FPI/FHBPI films can potentially be used as UV-shielding materials in harsh environments, such as in space. In particular, they could be employed to coat glass in spacesuits and capsules for protection against UV radiation exposure.

Another type of polyimides that were developed as promising materials for space applications are those containing diphenylphosphine oxide and trifluoromethyl side groups. These polymers were proposed for packaging materials of solar cells of low-Earth orbit (LEO) spacecrafts [41]. The side groups were selected in order to improve the solubility, optical transparency, and flame-retardancy of the polyimides without affecting their thermal stability [41,42]. The thermal stability of these films was evaluated before and after atomic oxygen (AO) exposure, through DSC and TG measurements performed in nitrogen atmosphere [41]. The DSC results unveiled a *T*_g_ value of 266 °C for the AO exposed film, with a decrease of 5 °C with respect to that of the film before exposure. The TG curves show a decomposition temperature (*T*_5%_) for the AO-exposed sample of 499 °C, which is 7 °C higher than the value detected for the non-exposed PI. The residual weight at 800 °C increased from 50.0 wt% to 51.6 wt%, and this can be ascribed to the minor oxidation caused by AO on the surface of the film. Overall, the DSC and TG results unveiled that the thermal stability of these phosphorus- and fluorine-containing PIs are not affected in a significant way by AO exposure, confirming these materials as good candidates for use in the solar cells of LEO spacecrafts.

**Figure 3:**
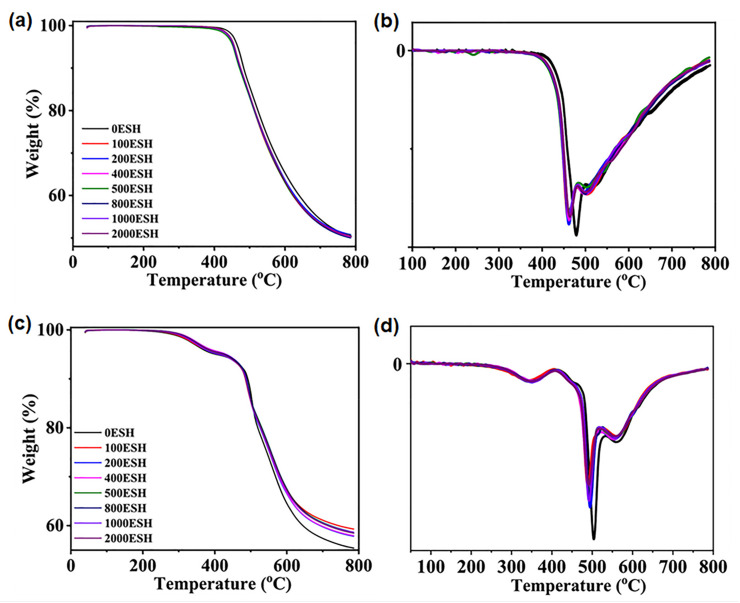
(**a**,**c**) TG curves and (**b**,**d**) TG rate of (**top**) CPI-L/Al and (**bottom**) CPI-T/Al films at different exposure doses (0–2000 ESH). Adapted with permission from Ref. [43].

Feng et al. considered some of the most used polyimides for space applications and investigated their performance in a simulated vacuum ultraviolet (VUV) environment [43]. The purpose of their work was to add detailed information about the behavior of these PI-based materials under space-like conditions. They realized aluminized colorless polyimide (CPI/Al) films that were tested under near-ultraviolet irradiation based on orbit equivalent solar hours (ESH). Experiments were carried out on two types of samples: CPI-L/Al and CPI-T/Al films with trifluoromethyl (-CF_3_) and alicyclic groups, respectively. The thermal stability of these materials was evaluated at different exposure doses using TG and DMA. The temperature at 5 wt% loss is identified as the onset degradation temperature (*T_onset_*) for the two types of films, and it was investigated using TG under nitrogen atmosphere. Before irradiation, the *T_onset_* of the CPI-L/Al and CPI-T/Al films was 450 °C and 471 °C, respectively. After 100 ESH irradiation, the *T_onset_* of the CPI-L/Al and CPI-T/Al films decreased by 3% and 5%, respectively. No significant variations were detected in the *T_onset_* for both films after increasing the exposure dose to 2000 ESH (Figure 3a,c). Considering the CPI-L/Al sample, a decomposition region was observed, and the maximum degradation rate was near 461 °C (Figure 3b). In the case of the CPI-T/Al film, two decomposition regions could be identified (Figure 3d), where the second one (near 250 °C) can be ascribed to the emission of small molecules [43,44]. The DMA measurements showed that the *T*_g_ of the CPI-L/Al and CPI-T/Al films before irradiation was 329 °C and 280 °C, respectively. This result can be related to the molecular structure flexibility of the CPI-T/Al film. After irradiation (0–2000 ESH), the *T_g_* values of the CPI-L/Al and CPI-T/Al films showed slight variations. The authors explained this behavior by hypothesizing that molecular chain breaking and cross-linking compensate for each other, leading to negligible changes in the *T*_g_.

Highly thermostable and transparent polyimides were synthesized using a semi-aromatic tetracarboxylic dianhydride (OHADA) [45]. The central benzene ring in this dianhydride confers higher thermal stability than other alicyclic dianhydrides, whereas the cyclohexane moieties inhibit the formation of CTCs on the resultant PIs. Two types of polyimides, indicated as OHADA-TFDB and OHADA-BAFL, were synthesized through a reaction between OHADA and 2,2′-bis(trifluoromethyl)benzidine, and between OHADA and 9,9-bis(4-aminophenyl)fluorene, respectively. The thermal stability of the OHADA-TFDB and OHADA-BAFL polyimides was studied using DSC, TG, and TMA. For the OHADA-TFDB samples, the TG results showed 1% mass loss at 482 °C and 5% and 10% weight loss at 537 °C and 547 °C, respectively. The char yield of OHADA-TFDB is above 57% at 800 °C. Concerning the OHADA-BAFL polyimide, the TG measurements resulted in 1% weight loss at 491 °C and 5% and 10% weight loss at 546 °C and 555 °C, respectively. The char yield of OHADA-BAFL is above 44% at 800 °C. Based on these results, the OHADA-TFDB polyimide shows slightly lower thermal stability than OHADA-BAFL. This can be ascribed to the flexible CF_3_ side groups of TFDB, and to the lower aromatic content with respect to OHADA-BAFL. In addition, the degradation temperature values are higher than those of PIs synthesized from wholly alicyclic dianhydrides [46]. The *T*_g_ values obtained from the DSC analysis are 339 and 385 °C for OHADA-TFDB and OHADA-BAFL, respectively. TMA was also used to measure the *T_g_* and to compare the values with other works in the literature using the same technique. The *T_g_* values from the TMA are 335 °C for OHADA-TFDB and 395 °C for OHA-DA-BAFL. Thus, the *T_g_* of OHADA-BAFL is close to the highest rank in the colorless PIs [47,48,49]. These results make the OHADA polyimides excellent candidates for advanced applications, such as those involving display substrates in the optoelectronic and aerospace industry.

### 2.2. Composites with Polyimide Matrix

Polyimide-based nanocomposites with silica filler (PI/SiO_2_) were developed as alternative materials for the glass cover sheets used in space solar cells [50]. Recently, Huang et al. produced PI/SiO_2_ nanocomposite films with different SiO_2_ contents through in situ polymerization, starting with fluorine-containing diamine (TFDB) and fluorine-containing dianhydride (6FDA) as monomers [51]. The fluorine-containing PIs showed remarkable light transmittance [52], whereas the filler was used to improve the radiation resistance and the thermal performances of the films. A silane coupling agent was used to avoid agglomeration of the SiO_2_ nanoparticles. The thermal stability of these PI/SiO_2_ films was tested using TG. The results from the analysis of the PI films with different SiO_2_ amounts showed that the decomposition temperature of the films increases with the increase in the filler loading. In particular, it was observed that for the neat polyimide (PI), and for the PI/SiO_2_ samples at 5 wt% (PIS05) and 10 wt% (PIS10), the thermal decomposition temperatures were 517, 522, and 525 °C, respectively. Moreover, the residual rate at 700 °C was higher with higher SiO_2_ loadings. This behavior can be ascribed to the excellent heat resistance of SiO_2_ [53,54]. The good dispersion of the SiO_2_ nanoparticles into the polymer matrix causes a dense crosslinked structure between the carbonyl group of the polyimide backbone and the silyl hydroxyl group of SiO_2_. This crosslinked structure inhibits the thermal vibration of the polyimide chains, thus improving the thermal stability of the nanocomposite with respect to the unfilled PI [51]. The silane coupling agent further hinders the movement of the polyimide chains. As the SiO_2_ content increases, the degree of cross-linking of the nanoparticles with the PI matrix is higher. Therefore, higher energy is required to break the chain during heating, resulting in the improved thermal stability of the PI/SiO_2_ films.

Nanocomposite films based on a highly fluorinated polyimide (FPI) and polydopamine (PDA) were fabricated to be used as UV shielding materials for space applications [55]. Polydopamine (PDA), also known as artificial melanin, was selected as an ultraviolet absorbent, whereas a highly fluorinated PI was selected as a matrix to reduce the color intensity and to increase the optical transparency of the FPI/PDA films. Several samples with a porous structure and different PDA contents were synthesized through hydrogen bonding between PDA and FPI, followed by cyclodehydration via chemical and thermal imidization. The porous structure results in a lower density of these materials and an increase in the UV propagation path. The properties of the FPI/PDA films were investigated using several techniques. The thermal stability was analyzed using TG, DMA, and DSC. The results from the TG unveiled that the presence of PDA improves the thermal stability of the pure FPI. In particular, for samples containing 1 wt% of PDA, the initial decomposition temperature, 5% decomposition temperature, and 10% decomposition temperature are as high as 462, 533, and 555 °C, respectively. The DMA measurements showed a maximum *T*_g_ value of 342 °C for the samples with 1 wt% of PDA, which is 26 °C above that of pure FPI. This can be explained by the presence of hydrogen bonds between the hydroxyl of PDA and the carbonyl of FPI, which has the effect of reducing the movement of FPI molecular linkage.

Tharakan et al. developed two novel flexible polyimides by reacting a new diamine containing two long/bulky aromatic pendant chains and flexible linkages, with tetra carboxylic acid dianhydrides [56]. In particular, aromatic dianhydrides such as 3,3′,4,4′-benzophenone tetra carboxylic acid dianhydride (BTDA) and 4,4′-(4,4′-isopropylidenediphenoxy) diphthalic anhydride (BPADA) were used to incorporate isopropylidene and keto groups into the polymer backbone. The resulting polyimides were used as matrices for embedding aromatic amine-functionalized silica, obtaining high-performance materials suitable for use in advanced applications, such as in space. These polyimides are not colorless because of the presence of several aromatic groups in the main chain and in the pendant chains. Nevertheless, they have good optical transparency, showing absorption of light at wavelengths shorter than 350 nm. The thermal stability of the resulting polyimides and of the nanocomposites with modified silica was analyzed through TG in nitrogen atmosphere (heating rate of 10 °C/min). The 10% decomposition temperature values are in the range of 364–482 °C, indicating good stability. Weight loss was observed in the range of 250–300 °C, which can be ascribed to the decomposition of the carbonyl and methylene groups. Above 300 °C, an inflection in the TG curves can be related to the breakage of the isopropyl group. In general, the aromatic groups and the ether linkages in the pendant groups ensure the high thermal stability of the polyimides. The presence of alkyl pendant groups causes a loss in thermal properties, but bulky aryl side chains with flexible linkages can effectively mitigate this effect. The nanocomposites realized in the work by Tharakan et al. exhibited higher thermal stability than the pure polyimides due to the presence of silica nanofillers [54]. In fact, the results showed that the decomposition temperature increases as the filler loading increases. Moreover, the functionalization of the silica nanopowder by the aromatic amino groups improves and preserves the rigidity and the thermal stability of the polymer matrix.

Recently, Zhang et al. developed transparent PI-based nanomaterials using a fluoro-containing PI and trisilanolphenyl-polyhedral oligomeric silsesquioxanes (TSP-POSS) as additives to improve the resistance to atomic oxygen (AO) degradation [57]. Different films were prepared, varying the amount of TSP-POSS between 0 and 25 wt%, and they were named 6FPI-0, 6FPI-5, 6FPI-10, 6FPI-15, 6FPI-20, and 6FPI-25 based on the filler content. The thermal stability of these films was evaluated using TG, DSC, and thermomechanical analysis (TMA), in view of their potential applications as spacecraft antenna substrates. The TG measurements unveiled that all of the PI films maintained their initial weights at temperatures above 450 °C, showing *T*_5%_ values in the range of 494–520 °C. The results also showed that the residual weight ratio at 760 °C for the PI films decreased in the following order: 6FPI-0 (53.8%) > 6FPI-5 (53.6%) > 6FPI-10 (48.4%) > 6FPI-15 (48.1%) > 6FPI-20 (47.4%) > 6FPI-25 (45.3%). This behavior can be ascribed to the formation of gaseous fluorine-silicon compounds derived from the reaction of decomposed fluoro species and silicon elements at high temperatures [57,58]. The DSC results unveiled that the *T_g_* values of the PI films containing TSP-POSS are quite similar, with small variations from 256.2 °C for the neat PI films to 260.2 °C for the films with 25 wt% of additives. Nevertheless, the TMA results revealed that the addition of TSP-POSS does affect the high-temperature dimensional stability of the PIs. Due to the plasticization effect of the TSP-POSS filler, the values of the coefficient of linear thermal expansion (CTE) for the composites are increased. Indeed, this effect could represent a limitation for the actual application of these materials as substrates for spacecraft antennas.

Liu et al. investigated the thermal behavior of glass/polyimide composites under the conduction condition with and without radiation [59]. Numerical simulations were used, and multi-scale unit cell models of PI, glass-fiber yarns, and composite materials were sequentially adopted. A finite element model was built using triangle and tetrahedral elements for 2D and 3D mesh formation, respectively. The results showed that the effective thermal conductivity of PI decreases with the increase in porosity, with a maximum of 39.5%. The transverse effective thermal conductivity of the glass-fiber yarns increases as the fiber volume fraction increases, with a maximum increasing ratio of 49.0%. Moreover, differences of 26.0% and 27.6% of the in-plane and out-of-plane effective thermal conductivities of the glass/PI composites were assessed with and without radiation. In particular, it was observed that under the condition of pure conduction (no radiation) the temperature affects the effective thermal conductivities of the composites to a negligible extent. In contrast, in the presence of radiation, the effective thermal conductivities increase with the increase in temperature. This might be due to the contribution of thermal radiation to heat transfer, which becomes more significant as the temperature rises. Overall, this research employed a simulation-based approach to explore the thermal behavior of composite materials, opening up new opportunities for their use in aerospace applications.

## 3. Thermal Stability of Carbon-Filled Polymer Composites for Space Applications

Over the past two decades, graphene nanoparticles (GNP) and carbon nanotubes (CNT) have been effectively used for improving polymer properties, including their thermal stability. Recently, specifically for space applications, novel carbon-filled polymer composites have been developed and their thermal properties have been opportunely investigated, with discussion of the role of the filler.

### 3.1. Structural Composites

Bel et al. developed poly(methyl methacrylate)/graphene (PMMA/GNP) nanocomposites suitable for applications on the outside of electronic devices in commercial satellites [60]. These materials showed good thermal resistance, lightness, and gamma and microwave absorption properties. In particular, the TG technique was used to investigate the thermal stability of these nanocomposites, and their behavior was compared with that of neat PMMA. Graphene nanoparticles with a surface area of 150 m^2^/g, thickness of 6 nm, and purity > 99.5% were supplied by Nanografi Nano Technology (Çankaya/Ankara, Turkey). The results showed that the addition of GNP at 0.25 wt% causes an increase in the 5% weight loss temperature (*T*_5%_) of neat PMMA, from 197 °C to 219 °C. Increasing the GNP content to 2 wt%, the *T*_5%_ reaches the value of 243 °C, confirming the enhanced thermal stability of these nanocomposites due to the presence of graphene [61]. Nevertheless, this improvement must be related to the good dispersion of the filler obtained through in situ polymerization. This process can lead to strong interfacial interactions between the PMMA matrix and the GNP filler, avoiding fractures, which can cause losses in terms of the mechanical properties and thermal stability [60].

Dey et al. studied the thermal behavior of nanocomposites based on a polybenzimidazole (PBI) polymer matrix filled with multi-walled carbon nanotubes (MWCNT), graphene oxide (GO), and hybrid GO-MWCNT reinforcement [62]. PBI was selected for its superior properties, such as chemical resistance in hostile environments, good fire resistance, and good mechanical properties. A modified Hummers’ method [63] was used to synthesize GO via chemical oxidation of the raw graphite powder. GO and MWCNT (purity 95%, diameter = 10–15 nm, length = 1–10 μm, density = 1.7 g/cm^3^) were used for preparing the PBI nanocomposites via an in-situ chemical polymerization approach. The authors compared the features of the neat PBI with those of GO/PBI, MWCNT/PBI, and GO-MWCNT/PBI nanocomposites in order to assess their potential use in aerospace structures and aerospace electronics, substituting traditional metallic materials. The thermal stability was evaluated using TG in nitrogen atmosphere. The results showed a two-step weight loss for the neat PBI: the first one below 415 °C, equal to 20.3%; the second one between 415 and 524 °C, equal to 25.1%. The first weight loss can be ascribed to the removal of moisture, absorbed by the sample during storage together with the decomposition of small molecules, whereas the second weight loss can be associated with the decomposition of the PBI macromolecules. By incorporating the carbon fillers into the PBI, the TG curves unveiled an enhancement in the thermal stability with respect to the pure PBI. In particular, higher temperatures of degradation onset (*T*_onset_) and higher temperatures of maximum mass loss (*T*_max_) were observed, with a reduction in the thermal decomposition rate. This can be explained by the carbon surfaces acting as scavengers of the free radicals derived from the heterolytic chain cleavage due to the thermal decomposition of the PBI. This effect results in a decrease in the thermal decomposition rate of the nanocomposites, and thus an increase in the degradation temperature. The thermal stability was further improved by incorporating the GO-MWCNT hybrid filler. In fact, in this case, the synergistic effect of GO and MWCNT causes a more effective heat dissipation within the nanocomposite, leading to increased thermal stability. As the GO-MWCNT loading increases (up to 10 wt%), the degradation temperatures (*T*_onset_ and *T*_max_) of the nanocomposites are higher, with an increase in the char yield. Moreover, this hybrid filler acts by mitigating the hygroscopicity of PBI, and by creating longer diffusion paths across the polymer. Therefore, the moisture barrier performance of the nanocomposites is improved. This effect also contributes to the enhanced thermal stability of the hybrid GO-MWCNT/PBI nanocomposites.

### 3.2. Functional Composites

Recently, novel functional polymer-based composites have been developed to withstand the extreme conditions of space while performing critical functions to ensure the safety and success of space missions [64,65]. These composites serve specific functions that are crucial for spacecraft, satellites, and space exploration. In this context, the assessment of the thermal behavior of these materials plays a crucial role.

Polymers such as organosilicon elastomers (silicones) have been extensively investigated, demonstrating their remarkable thermal resistance capabilities [66,67,68]. Zhang et al. performed a numerical study on the heat transfer behavior of polydimethylsiloxane (PDMS)-based materials filled with graphene foam (GF) [69]. The simulation of heat transportation was carried out considering the interfacial thermal conductance, the contact thermal resistance, and the geometry and volume fraction of graphene foam. A finite element model was used, unveiling that the interfacial thermal conductance between silicon and filler has a negligible effect on the thermal conductivity of the composite. Overall, the results suggested that the use of GF with short struts and a large effective radius can enhance the thermal conductivity of these materials.

At present, silicon rubbers, polyurethane, and polyethylene are widely used to fabricate functional carbon-filled composites suitable for space applications, such as radiation monitoring and radiation shielding [65,70,71,72,73]. The specific applications are described in the following subsections.

#### 3.2.1. Composites for Space Radiation Monitoring

**Figure 4:**
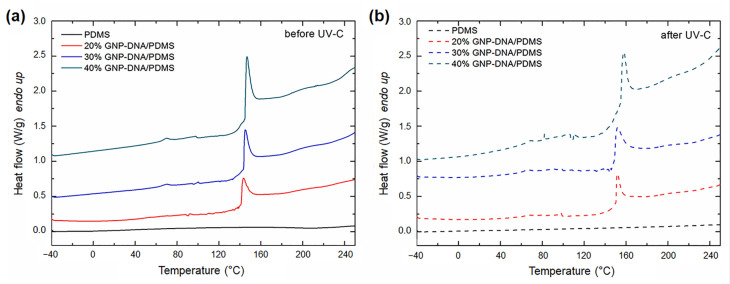
DSC thermograms of GNP-DNA/PDMS films with different content of GNP-DNA filler (**a**) before and (**b**) after UV-C exposure (8 days, irradiance 6.3 W/m^2^). Adapted with permission from Ref. [76].

Polymer-matrix nanocomposites loaded with graphene-deoxyribonucleic acid (GNP-DNA) fillers were fabricated for potential application in space radiation monitoring systems [65,74,75,76,77,78]. Graphene nanoplatelets (grade AO-4) with a surface area ≤ 40 m^2^/g, purity of 98.5%, average flake thickness of 60 nm, and a particle lateral size ≤ 7 µm were supplied by Graphene Supermarket (Graphene Laboratories Inc., Ronkonkoma, NY, USA). GNP and double-stranded DNA (protein content ≤ 1%, A_260nm_/A_280nm_ ≥ 1.5) were combined to form a hybrid nanomaterial using ultrasound sonication. The modification of GNP with DNA enabled the creation of a radiation-sensitive nanofiller, which was opportunely incorporated into the polymer matrix. The thermal behavior of the composites based on GNP-DNA fillers embedded in a polydimethylsiloxane (PDMS) matrix was extensively investigated [75,76,77,78]. In particular, GNP-DNA/PDMS films were exposed to UV-C and their properties were examined before and after irradiation [76]. The samples were exposed to a UV-C irradiance of 6.3 W/m^2^ over 8 days, with a maximum radiation dose of 4.4 × 10^6^ J/m^2^. DSC analysis was performed in a wide temperature range (from −40 to 250 °C) for evaluating the thermal behavior of the films at different GNP-DNA contents (20, 30, 40 wt%). Figure 4 reports the DSC curves of the nanocomposites in comparison with neat PDMS.

The samples filled with GNP-DNA showed an endothermic peak at about 146 °C (Figure 4a), which shifted to higher temperatures after irradiation (Figure 4b). The neat PDMS film exhibited thermal stability over the entire temperature range of analysis, before and after UV-C exposure. The nature of the endothermic peak was explained by the authors after studying the thermal behavior of pure DNA at the same conditions used for the nanocomposite samples. Indeed, the presence of the peak for the nanocomposites can be ascribed to the thermal degradation of the DNA component. Other results from the literature confirm that the degradation temperature of dry DNA (powder form) is higher than those measured for DNA in aqueous solution (between 50 and 90 °C) and can reach values of up to 170 °C [79]. The shift of the peak after radiation exposure was ascribed to a UV-induced crosslinking of the DNA chains, which increases the thermal stability of the biological component. It was also observed that the enthalpy change (ΔH) associated with the endothermic event increases with the loading of the GNP-DNA filler, and hence with the content of DNA. After UV-C irradiation, all of the samples containing DNA showed a shift of the denaturation peak and a corresponding increase in the Δ*H* value, which is a further sign of the higher cross-linked nature of DNA.

In subsequent works, Toto et al. investigated the properties of self-standing GNP-DNA/PDMS and GNP/PDMS nanocomposites before and after exposure to space environment conditions [77,78]. GNP-DNA/PDMS, GNP/PDMS, and neat PDMS samples were exposed to solar radiation in a high-vacuum (HV) environmental chamber specifically designed to simulate the space environment [80]. The thermal behavior of these composites was analyzed through DSC in the temperature range of −40 to 200 °C before and after 24 h testing under HV (~10^−6^ mbar) and HV combined with solar irradiation (4 h) [77]. The results revealed an endothermic peak at about 148 °C only for the samples containing the DNA-modified GNP filler, confirming the results of the previous study [76]. For the GNP-DNA/PDMS nanocomposites, the experiments in HV without solar irradiation unveiled that the thermal stability of the DNA did not change significantly. In contrast, after testing under HV combined with solar irradiation, the DNA decomposition peak shifted to larger values (167.1 °C), and the corresponding Δ*H* decreased from 38.7 J/g to 22.4 J/g. These variations can be ascribed to DNA crosslinking via reactive oxygen species, which are generated by the combined effect of UV and blue light radiation [81,82]. The formation of DNA-PDMS crosslinks improves the thermal stability of the GNP-DNA/PDMS nanocomposites, with a shift of the DNA decomposition peak to higher temperatures.

#### 3.2.2. Composites for Space Radiation Shielding

**Figure 5:**
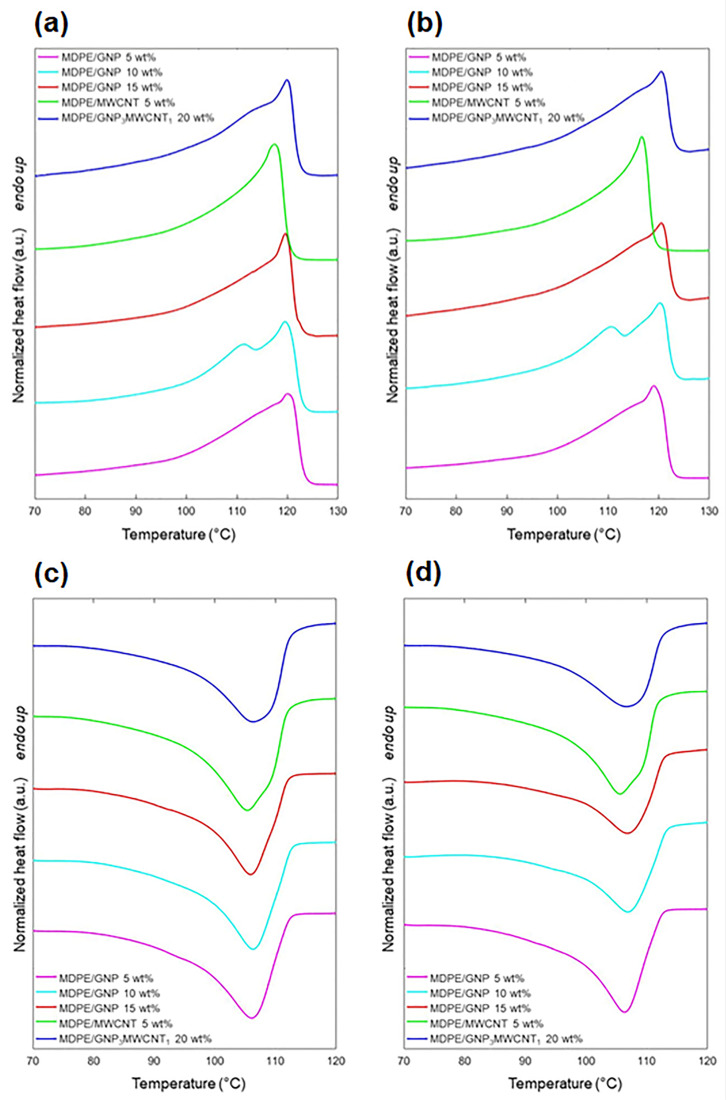
DSC thermograms upon (**a**,**b**) heating and (**c**,**d**) cooling for MDPE/GNP 5 wt%, MDPE/GNP 10 wt%, MDPE/GNP 15 wt%, MDPE/MWCNT 5 wt% and MDPE/GNP_3_MWCNT_1_ 20 wt% nanocomposites (**a**,**c**) before and (**b**,**d**) after proton irradiation. Adapted with permission from Ref. [70].

For potential applications in the space environment, carbon-based nanocomposites made of medium-density polyethylene (MDPE) loaded with multi-walled carbon nanotubes (MWCNT), GNP, and hybrid MWCNT/GNP fillers have been analyzed before and after proton irradiation [70]. Exfoliated GNP (grade C750) with a thickness of ~2 nm, average diameter of <2 μm, and specific surface area of ~750 m^2^/g were supplied by XG Sciences (Lansing, MI, USA). Pristine MWCNT with an outer diameter of ~9.5 nm, average length of ~1.5 μm, and specific surface area of ~250–300 m^2^/g were purchased from Nanocyl S.A. (Sambreville, Belgium). The results were used to evaluate the potential application of these materials for radiation shielding applications. The thermal behavior of the MDPE/carbon nanocomposites was investigated through DSC. In particular, the samples were analyzed before and after proton exposure in the temperature range of −45 to 150 °C with heating and cooling rates of 10 °C/min, and the obtained thermograms are reported in Figure 5. The results highlight slight differences in the values of the melting (*T*_m_) and crystallization (*T_c_*) temperatures among the different nanocomposites and after the radiation exposure. Starting from the DSC curves, the degree of crystallinity (*X_c_*) was calculated for each sample. It was observed that, in the presence of GNP, irradiation caused a decrease in the crystallinity. The crystallization enthalpy (Δ*H*_c_) decreases after irradiation due to the formation of cross-links and chain branches that hinder chain mobility during the crystallization process, leading to the formation of imperfect lamellae [83]. In addition, a slight decrease in *X_c_* was observed as the GNP content increased. This result can be related to the tendency of GNP to hinder the molecular mobility of the matrix at relatively high filler concentrations (above 3–5 wt%) [84,85], thus affecting the growth of crystallites [85]. In the case of the MDPE/MWCNT 5 wt% samples, the crystallinity was slightly higher than for the MDPE/GNP 5 wt% nanocomposites. This is because the GNP filler imposes more constraints around the polymer chains, inducing a greater fraction of polymer chains to be trapped in the graphene network [86]. All of the nanocomposites showed a decrease in *X_c_* after proton irradiation, which was negligible for the MDPE/MWCNT 5 wt% samples. The results indicate that the MDPE/MWCNT 5 wt% nanocomposite is a good candidate for radiation shielding applications due to the negligible variations observed in its physico-chemical properties after proton exposure.

Several works have focused on the thermal stability of carbon-filled polymer composites for electromagnetic interference (EMI) shielding in the space environment [71,72,73]. Among these, composites based on a polyurethane (PU) matrix were tested [71]. PU was used as a foam as its porous structure provides the advantage of matching the impedance between the shielding material and the ambient environment. In particular, polyurethane was filled with different amounts of graphite (G) using an in-situ preparation method, obtaining homogeneous PU/G composite foams. The physico-chemical properties and the shielding efficacy (SE) of these materials were tested to establish their suitability for EMI shielding applications in space. The thermal behavior was evaluated using TG with sample heating from room temperature to 600 °C at a heating rate of 10 °C/min, under nitrogen atmosphere. The TG curves showed that the thermal stability of the PU was slightly improved by the addition of graphite, whereas the T_5%_ increased from 196 to 217 °C in the presence of the filler. The shift of the T_5%_ value can be ascribed to the restrictions on the thermal motion of the polymer chain segments caused by the presence of graphite. The temperature at which the maximum decomposition rate occurred (*T*_max_) for the neat PU and the composites at 5 and 30 wt% of filler loading was found to be 317, 335, and 355 °C, respectively. These results confirm that the carbon filler can increase the thermal stability of polyurethane. The decomposition reactions that occurred in the temperature range of 200–400 °C can be justified by the depolymerization of polyurethane chains and the release of precursors, such as polyols and isocyanates [87]. Next, the isocyanates are dimerized to form carbodiimides, and the volatilization of small-molecule compounds occurs. The depolymerization is followed by dehydration and polyether formation. Last, the second degradation stage appeared in the range 350–600 °C, and this is due to the degradation of the precursors originating from the reaction of carbodiimide with alcohol or water.

Among materials with EMI shielding properties, Dun et al. developed composite foams based on a poly(vinylidene fluoride) (PVDF) matrix filled with carbon nanotubes using a solid-state supercritical CO_2_ foaming strategy [72]. PVDF was selected for its chemical resistance, UV resistance, thermal stability, flame retardancy, and dielectric properties [88], whereas MWCNT were used for improving the mechanical, electrical, and EMI shielding properties. Carbon nanotubes (grade Flotube 9000) prepared through catalytic chemical vapor deposition (CCVD), with a length of 1–10 µm and diameter of 10–15 nm, were supplied by Nanografi Nano Technology (Çankaya/Ankara, Turkey). The PVDF/MWCNT composite foams were tested using different techniques to assess their potential use as EMI shielding materials in aircraft and spacecraft applications. In particular, the thermal properties of these materials were investigated to improve the foaming process. Measurements were carried out using regular DSC and HP-DSC under a high CO_2_ pressure of 45 bar. Samples were equilibrated at 200 °C and then isothermally held for 5 min to remove the thermal history. Subsequently, the samples were cooled down to 30 °C at a rate of 10 °C/min. After holding for 5 min at 30 °C, the samples were heated up to 200 °C at the same rate. The crystallinity degree calculated from the regular DSC and HP-DSC thermograms slightly decreased with the increase in the MWCNT loading, meaning that the high melt viscosity limited the PVDF molecular chain movement for crystal growth. Comparing the results from the regular DSC and HP-DSC, at the same loading of the MWCNT, the crystallization peak temperature from the HP-DSC was a little lower than the corresponding temperature detected by regular DSC. In addition, the X_c_ from the HP-DSC was markedly lower than the X_c_ from the regular DSC. At high CO_2_ pressure, an improvement in the PVDF molecular movement may have favored crystal growth and thus the presence of more crystal nuclei. Hence, the increase in entangled PVDF molecules and in more imperfect crystalline structures lead to a low crystallization degree [89]. Moreover, both the regular DSC and HP-DSC showed melting peak temperatures that decreased with the increase in the MWCNT loadings. This can be related to the formation of imperfect crystals due to the heterogeneous nucleation effect of carbon nanotubes [90].

Na et al. fabricated lightweight and flexible composite films with excellent thermal stability and suitable mechanical properties for use as high-performance EMI shielding materials in hostile environments, such as in space [73]. These films were fabricated using a poly(ether ether ketone) (PEEK) matrix and multi-walled carbon nanotubes wrapped by poly(ether sulfone) (wrapped MWCNT) as conductive fillers. MWCNT grown through chemical vapor deposition (CVD), with an outside diameter of 10–20 nm, length of 10–30 μm, and purity of >95% were supplied by Chengdu Organic Chemicals Co., Ltd. (Chengdu, China). GENIOPLAST^®^ PELLET S (GPPS, high-temperature lubricant) was used as a processing additive. GPPS reduces the PEEK melt viscosity and improves the dispersion of the wrapped MWCNT. TG measurements were performed to investigate the thermal stability of these materials. The results showed that GPPS does not affect the thermal stability of the PEEK/MWCNT composite: samples with the lubricant have a temperature of 5% mass loss (*T*_5%_) of about 595 °C, close to that of the PEEK/MWCNT composites without GPPS. TG measurements were also performed on composite films with different loadings of wrapped MWCNT. In this case, with the increase in MWCNT loadings, a shift of the initial decomposition curves to higher temperatures was observed. This is due to the high thermal stability of the MWCNT [91], with a decomposition temperature greater than 600 °C, exceeding that of neat PEEK. Moreover, the wrapped MWCNT create a network structure in the polymer matrix, which hinders the thermal motion of the PEEK chains. Overall, this results in higher thermal stability with respect to the unloaded PEEK. In particular, the best results were obtained for the PEEK/MWCNT composite films with 9 wt% of wrapped nanotubes, showing a *T*_5%_ higher than 586 °C.

The thermal behavior of the polymer-based composites described above for specific aerospace applications is summarized in Table 1.

## 4. Thermal Stability of Epoxy Composites Tailored for Aeronautical and Aerospace Applications

Epoxy composites, a composite material class with a thermosetting matrix, boast impressive mechanical strength, stiffness, and corrosion resistance. These composites exhibit high-performance characteristics and find wide application in the aircraft and aerospace industries. It is essential to underscore that, for any given application, the meticulous selection of an appropriate epoxy-hardener system and factors such as suitable process variables (curing time and temperature), stoichiometric ratio, degree of crosslinking, and choice of reinforcing filler used are fundamental in achieving the desired functional and/or structural properties for the developed epoxy composites [92,93,94,95,96,97,98]. In this regard, in order to counteract the electrical insulating property and poor flame resistance of epoxies that impregnate carbon fabric, a multifunctional epoxy formulation that was effectively designed and used to impregnate carbon fabric was developed by Guadagno et al. [97]. The multifunctional panel reinforced with carbon fibers was produced through bulk infusion—a modified resin film infusion process. To impart the resin electrical conductivity, a quantity of 0.5 wt% of Multi-Wall Carbon Nanotubes (MWCNTs) was dispersed in the resin, while to increase the flame resistance, a quantity of 5 wt% of Glycidil Polyhedral Oligomeric Silsesquioxanes (GPOSS) was solubilized in the epoxy mixture. In addition, a mixture of 4,4′-diaminodiphenylsulfone (DDS) (53.4 wt%) and bis(3-aminophenyl)methylphosphine oxide (BAMPO) (46.7 wt%) was used as a curing agent. The electrical conductivity values were found to be sufficiently satisfactory, being 4.02 × 10^−2^ S/m for the multifunctional resin and 1.39 × 10^4^ S/m for the conductivity in the plane of the panel, while the value of the Limiting Oxygen Index (LOI) of the multifunctional resin was found to increase from 27 to 36%. The low-frequency vibration damping and acoustic insulation characteristics of the manufactured panel were also evaluated. The results demonstrated improved efficiency over a baseline configuration, featuring nearly double the modal damping and a 10 dB gain in overall noise reduction. Furthermore, through good correlation between the computational results achieved at the molecular scale and the experimental results obtained through direct current (DC) measurements and the electrical mapping of conductive nanodomains in hybrid systems through Tunneling Atomic Force Microscopy (TUNA), Raimondo et al. [99] demonstrated the synergistic effect of the two conductive nanofillers—Multi-Wall Carbon Nanotubes (MWCNTs) and Graphene Nanosheets (GNs)—on the electrical properties of nanohybrid epoxy systems. The TUNA technique proved to be extremely effective for understanding the synergistic effect of the two nanofillers on the interface performance of hybrid composites, thus enabling a breakthrough in a research area where the analysis of this aspect is still very incomplete. It is noteworthy that by adding a minimal amount of graphene nanosheets (GNs), the synergistic effect was recorded for the hybrid composites at 0.1 wt% filler mix. In particular, for this low hybrid nanofiller concentration, a particularly interesting synergy towards an increase in the electrical conductivity of several orders of magnitude and a lowering of the Electrical Percolation Threshold (EPT) was detectable.

Both the computational and experimental results highlight that, owing to the hybrid MWCNT/GNs network formation, the hybrid nanocomposites outperform their single-nanofiller counterparts.

The exceptional electrical performance manifested by hybrid epoxy systems are due to π–π bond interactions between the Multi-Wall Carbon Nanotubes and the Graphene Nanosheets dispersed in the hosting epoxy resin.

Owing to numerous advantages surpassing conventional materials like metals or ceramics [15,100,101,102,103,104,105,106,107,108], epoxy composites are increasingly playing a primary role in the aeronautical and aerospace sectors. Some of their pivotal benefits include [109,110,111,112]:

- Exceptional strength-to-weight ratio, giving them a fusion of robustness and lightness, thus minimizing the overall weight and fuel consumption of aircrafts.
- Remarkable creep resistance, allowing them to stave off deformation under persistent stress or high temperatures.
- High tensile strength at high temperatures, enabling them to endure considerable thermal stresses without fracturing.
- Excellent adhesiveness, ensuring compatibility with various materials while facilitating complex shape formation.
- Exceptional resistance to heat and solvents, enabling them to resist the corrosion and degradation caused by chemical or environmental factors.
- Extended lifespan, which can be lengthen the service life of aircrafts with a reduction in maintenance costs.

However, epoxy composites also have drawbacks [109,113,114,115,116] in the context of aeronautics and aerospace:

- Need for higher temperature treatments than unfilled resins to reach the same curing degree, which may increase the energy consumption and cost of fabrication.
- Difficult to be recycled or disposed of due to their infusible and insoluble nature, which may pose environmental and health hazards.
- Susceptibility to degradation upon exposure to UV radiation or moisture that may compromise their mechanical and electrical properties and service life.
- Constrained availability or prohibitive costs compared to alternative materials, which may obstruct their widespread use and affordability.
- Greater immunity to electricity, which exacerbates the risk of electrostatic discharge or lightning strike damage.
- Lower thermal conductivity, which may cause thermal expansion mismatches or overheating issues.
- Reduced resistance to impacts, which may cause delamination or fractures under high stress or fatigue loading.

Driven by an unrelenting ambition for progress in the aeronautical and aerospace industries, manufacturers tirelessly seek winning strategies to enhance the performance of commercial and military aircrafts, leading them to develop materials specifically designed to ensure awesome structural and/or functional properties. Composite materials have undoubtedly emerged as essential contributors to the current and future aerospace components, primarily due to their astonishing strength and stiffness-to-weight ratio and superior physical properties.

In advanced aeronautical and aerospace applications, epoxy thermal stability ensures that epoxy resins maintain their mechanical and electrical properties despite exposure to high temperatures and thermal stresses. Thermal stability remains critical for aerospace materials, which must withstand extreme conditions such as high-speed aerodynamic heating, solar radiation, or atmospheric re-entry. The decomposition of epoxy composites can result in deteriorated strength, stiffness, ruggedness, and dimensional stability. Therefore, fire resistance is a vital safety prerequisite for aerospace materials. The thermal stability of epoxies relies considerably upon the chemical structure of the resin, the curing agent, the crosslink density, and the incorporation of additives or nanofillers [114,117]. Many and varied factors influence the thermal stability of epoxy composites, such as:

- The type, shape, and particle size of the filler [118,119]: different fillers have different thermal properties and can affect the heat transfer and thermal expansion of the composite.
- The dispersion state of the filler in the matrix [118,119]: a uniform dispersion of filler can improve the thermal conductivity and reduce the thermal degradation of the composite.
- The interfacial interaction between filler and matrix [118,119]: strong interfacial bonding can enhance the mechanical and thermal properties of the composite and prevent filler agglomeration and matrix cracking.
- The orientation degree of the fillers in the matrix [118,119]: a high degree of orientation can increase the thermal conductivity and stability of the composite along the direction of orientation.
- The chemical composition of the filler and the matrix [120,121]: some fillers, such as boron nitride nanosheets, can improve the thermal stability of epoxy composites by forming a protective layer on the surface or reacting with oxygen to reduce flammability [118,119,120].

**Figure 6:**
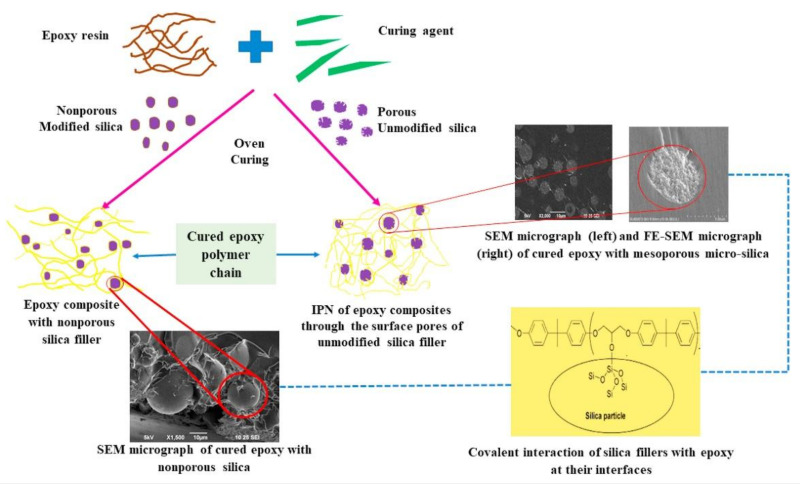
Illustrative diagram of the process of obtaining an epoxy polymer resin composite by incorporating nano/micro silica filler [120].

**Figure 7:**
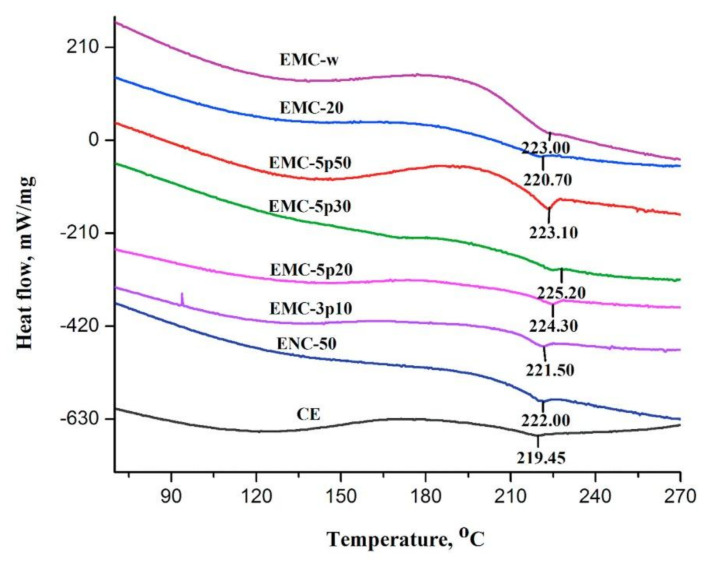
DSC curves of unfilled epoxy resin and epoxy-silica nano/micro-composites [120].

**Figure 8:**
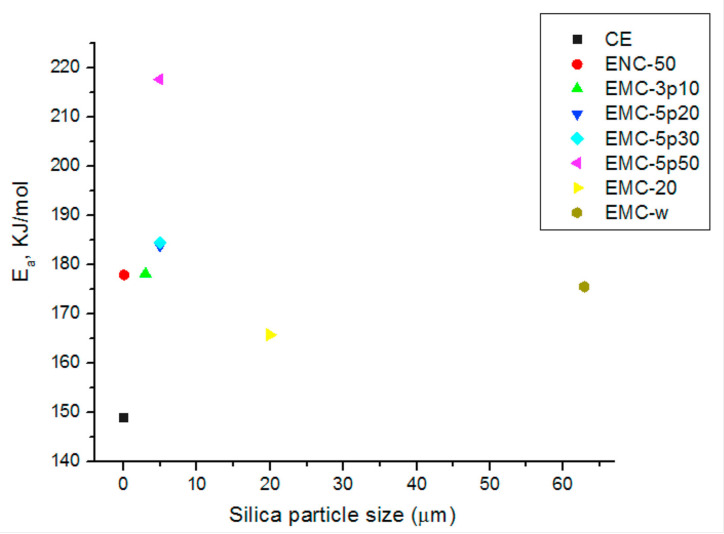
Average activation energy (E_a_) graph for thermal degradation of the epoxy-silica composites varying with particle size of silica fillers [120].

In striving to transcend the constraints linked to the thermal stability of epoxy composites, innovative epoxy blends have been formulated for utilization in the aeronautics and aerospace industries. In particular, certain studies have disclosed that the integration of mesoporous silica micro-filler [120] or microencapsulated phase change materials (MPCM) [122] within epoxy resin has the potential to enhance both its thermal stability and mechanical properties. The primary objective of this research work [120] was to improve the thermal stability of DGEBA epoxy resin by adding mesoporous silica micro-filler (5 μm, pore size = 50 nm) as a reinforcement material. Figure 6 shows the illustrative diagram of the process of obtaining an epoxy polymer resin composite by incorporating nano/micro silica filler. The authors compared the thermal properties of the composite with other types of silica fillers, such as nano-silica, non-porous micro-silica, and irregular micro-silica. Their findings revealed that the mesoporous silica composite exhibited the highest glass transition temperature (Figure 7) and activation energy for thermal degradation among all the samples (Figure 8). Indeed, as depicted in Figure 8, an average activation energy value of 148.86 kJ/mol was recorded for the thermal degradation of unfilled epoxy resin (CE). The maximum activation energy range was instead recorded for composites loaded with mesoporous microsilica. The EMC-5p50 sample showed the highest mean value of 217.6 kJ/mol. This remarkable enhancement was ascribed to the polymer invading the silica pores and forging formidable interfacial bonds. The study successfully demonstrated a straightforward yet efficacious methodology for fabricating epoxy composites boasting sophisticated thermal stability courtesy of the mesoporous silica micro-fillers.

**Figure 9:**
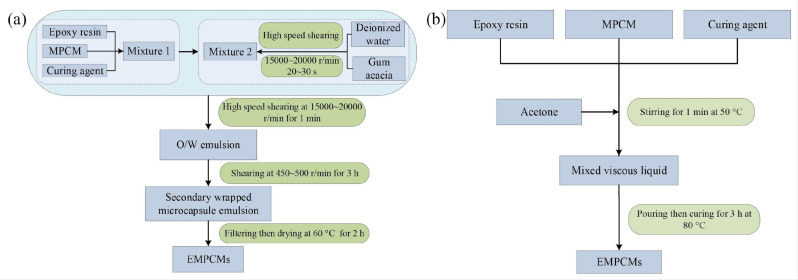
Preparation of EMPCMs: (**a**) secondary wrapping method; (**b**) pouring method. Reproduced with permission from Ref. [122].

The main goal of the research article [122] was to improve the thermal stability and mechanical properties of microencapsulated phase change materials (MPCM) by compounding them with epoxy resin. A comparative analysis of two compounding methods—the secondary wrapping method and the pouring method (Figure 9)—revealed that the pouring method yielded superior results.

**Figure 10:**
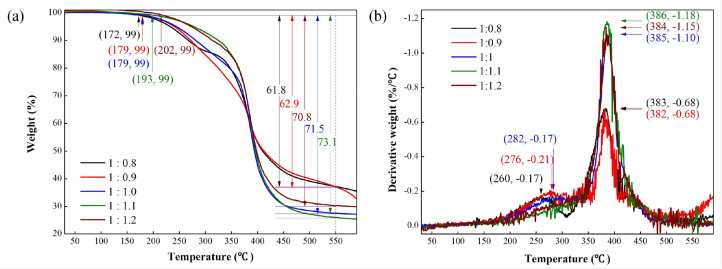
TG and DTG thermograms of EMPCMs wrapped with different epoxy resins: (**a**) TG; (**b**) DTG. Reproduced with permission from Ref. [122].

Moreover, the authors delved into examining the diverse types and quantities of epoxy resin and their impacts on the properties of composite phase change materials (EMPCMs). A variety of analytical tools—including TG, DSC, scanning electron microscopy (SEM), and heat treatment analysis—were employed to elucidate these effects. Conclusively, they discovered that epoxy resin significantly bolstered the thermal stability (Figure 10) and mechanical properties of MPCM by forming robust interfacial bonds. However, striking an equilibrium between phase change enthalpy and compressive strength remained crucial in determining the optimal type and content of epoxy resin for EMPCMs. It was surmised that bisphenol A epoxy resin (EP3) at a concentration of 50 wt% demonstrated superior efficacy for EMPCMs, owing to its high enthalpy, compressive strength, and crush value, thus rendering it compatible with asphalt pavement applications.

In order to overcome the limitations related to the thermal stability of epoxy composites, in the last few years, various nanofillers have been explored to reinforce epoxy composites, such as carbon nanotubes (CNTs), graphene derivatives, and inorganic 2D nanomaterials [111]. Guadagno et al. [113] describe the development and characterization of composite materials based on nanofilled epoxy resins envisioned for crafting structural aeronautical components that impart optimal lightning strike protection due to the enhanced electrical conductivity borne by the incorporation of carbon nanostructured forms, such as multi-wall carbon nanotubes (MWCNTs) or heat-treated carbon nanofibers (CNFs). This paper also investigates the electrical, mechanical, and thermal properties of the composites using different techniques. This paper demonstrates that the nanofillers improve the electrical conductivity, glass transition temperature, and fracture toughness of the epoxy composites.

Graphene emerges as one of the most prominent 2D nanofillers for epoxy composites due to its high aspect ratio, specific surface area, mechanical strength, and stiffness. Other 2D nanomaterials, such as transition metal dichalcogenides (TMDs), hexagonal boron nitride (hBN), and calcium silicate, have also been studied for enhancing the mechanical and thermal properties of epoxy composites [109,111]. However, there are still some challenges associated with the use of 2D nanofillers for epoxy composites, such as dispersion issues, the lack of standardization, health and environmental hazards, and scalability. Therefore, further research is needed to address these challenges and optimize the design and fabrication of 2D nanofilled epoxy composites for aerospace applications [111].

The thermal stability of epoxy composites in aeronautical and aerospace applications can be assessed using several sophisticated methods. In particular:

- TG analysis quantifies the variation of the composite’s mass loss as a function of the temperature or time under controlled conditions, offering insight into the onset temperature, maximum degradation rate, activation energy, and char yield of the thermal decomposition process [120,122,123].
- DSC analysis: by measuring the variation of heat flow into or out of the composite with the temperature or time in a controlled environment, this method provides valuable data on the glass transition temperature, curing degree, crystallization behavior, and specific heat capacity of the composite [120,122].
- SEM technique: by providing images of the surface morphology and microstructure of the composite before and after thermal exposure, this method enables the evaluation of the dispersion, distribution, and interaction of the nanofillers with the epoxy matrix. It can also provide information on damage mechanisms such as cracking, delamination, or char formation [120,122,123].
- According to some sources [109,124], the thermal stability of epoxy composites for aeronautical applications can be assessed through the use of TG measurements coupled with gas analyzers, such as Fourier transformed infrared spectroscopy (FTIR) or mass spectroscopy (MS) techniques. These techniques enable the analysis of the gases evolved during the thermal decomposition of the composites and to characterize their decomposition mechanism [124]. Another way to enhance the thermal stability and fire resistance properties of epoxy composites is to coat them with nanosized calcium silicate-reinforced polybenzimidazole composite [109] or to reinforce them with 2D nanomaterials such as graphene, transition metal dichalcogenides (TMDs), hexagonal boron nitride (hBN), and their hybrids [111].
- Thermomechanical analysis (TMA): this method measures the dimensional change of a sample as a function of the temperature or time under a controlled atmosphere and load. It can provide information about the thermal expansion coefficient, softening temperature, and creep behavior of the composite [125].
- Dynamic mechanical analysis (DMA): this method measures the mechanical response of a sample as a function of the temperature or time under a controlled atmosphere and oscillatory load. It can provide information about the viscoelastic properties, storage modulus, loss modulus, and damping factor of the composite [125].

Numerous experimental parameters can be employed to assess the thermal stability of epoxy composites, including:

- The mass loss of the composite as a function of the temperature or time, which indicates the degree of thermal degradation and decomposition [120,126].
- The *T*_g_ value of the composite, which indicates the temperature at which the composite changes from a glassy state to a rubbery state [120,126].
- The activation energy of the thermal degradation process, which indicates the energy barrier for the decomposition reaction, thus representing a sort of thermal stability parameter of the composite [120].
- The residual mass of the composite after thermal degradation, which indicates the amount of char or ash formed and the flame retardancy of the composite [120,126].

Epoxy composites find extensive applications throughout aeronautics for various structural and engineering purposes, such as fuselages, wings, propellers, and landing gears. However, these applications also expose the composites to harsh thermal environments, such as high temperatures, thermal shocks, thermal cycling, and fire. Therefore, epoxy composites must possess high thermal stability to withstand these conditions and maintain their mechanical and functional properties.

Unique thermal stability prerequisites for aeronautical epoxy composites comprise include:

- High *T*_g_ value: above *T*_g_, the epoxy matrix loses its stiffness and strength and becomes more susceptible to creep and fatigue. Therefore, epoxy composites must have a high *T_g_* to operate at elevated temperatures without compromising their performance [109,120,127].
- Low thermal expansion coefficient (CTE): this is the measure of how much the composite expands or contracts when heated or cooled. A high CTE can cause thermal stresses and strains in the composite due to the mismatch between the CTE of the epoxy matrix and the fiber reinforcement. This can lead to cracking, delamination, and failure of the composite. Therefore, epoxy composites must have a low CTE to minimize thermal deformation and damage [109,120,127].
- High thermal conductivity: this is the measure of how well the composite can transfer heat within its structure. High thermal conductivity can help dissipate heat from the composite and prevent overheating and degradation. Therefore, epoxy composites must have a high thermal conductivity to enhance their heat dissipation and cooling efficiency [109,120,128].
- High flame retardancy: this is the measure of how well the composite can resist ignition and combustion when exposed to fire. A high flame retardancy can help prevent or delay the onset of fire and reduce the flammability and smoke emission of the composite. Therefore, epoxy composites must have a high flame retardancy to improve their fire safety and survivability [109,120,128]. On the other hand, although bromine-based materials, as well as those including antimony oxide, are relevant efficient flame retardants [129,130], attention is paid to monitor the amount of bromine [129] and to try to keep its content as low as possible in the effort to develop more environment-friendly systems.

Various methods can be employed to increase the glass transition temperature (*T*_g_) of epoxy composites, including the following:

- Choosing a suitable epoxy resin and curing agent system: different epoxy resins and curing agents have different chemical structures and functionalities that affect the degree of crosslinking and the mobility of the polymer chains. In general, a higher degree of crosslinking and a lower mobility result in a higher *T*_g_. Therefore, epoxy composites must use a resin and a curing agent that can form a highly crosslinked and rigid network [128,131,132].
- Optimizing the curing conditions: the curing conditions, such as the temperature, time, and pressure, affect the degree of conversion and the quality of the cross-linked network. A higher degree of conversion and a smaller number of defects or voids lead to a higher *T*_g_. Therefore, epoxy composites must be solidified under the optimal conditions that can ensure a complete and uniform reaction [128,131,132].
- Adding modifiers or fillers: modifiers or fillers can be added to the epoxy system to modulate its properties, such as its toughness, thermal conductivity, flame retardancy, etc. However, some modifiers or fillers can also affect the *T*_g_ of the epoxy composite by changing its molecular structure, intermolecular interactions, or free volume. Therefore, epoxy composites must use modifiers or fillers that can increase or maintain the *T*_g_ of the epoxy system [128,131].

## 5. Conclusions

Underlying the conceptualization of this review paper is the key role played by thermal stability for high-performance properties in the aeronautical and space fields. In fact, in this review paper, through the use of recent results, an overview on the thermal stability evaluation of polymers and polymer-based composites tailored for advanced applications has been carried out. Thermal stability is the ability of a material to maintain its properties and structure when exposed to high temperatures. It is very crucial in this context because the materials used in aeronautical and space applications are subjected to extreme thermal environments—such as re-entry, combustion, propulsion, etc.—that can cause thermal stress, deformation, degradation, or failure of the materials. Therefore, the materials need to have high thermal efficiency to reduce the energy consumption, fuel cost, and greenhouse gas emissions, as well as to improve the performance and durability of the systems. They also require high thermal adaptability to cope with the varying thermal conditions—such as solar radiation, planetary albedo, infrared flux, deep space coldness—that can affect the thermal balance and control of the systems.

Therefore, thermal stability is a key requirement for the selection, design, and development of materials for aeronautical and space applications. In this regard, we have investigated the thermal stability of polyimide-based materials, carbon-filled polymer composites, and epoxy composites. Polyimide-based materials are polymers that have excellent thermal stability, mechanical strength, electrical insulation, and radiation resistance. They can be used as coatings, films, adhesives, and composites for various purposes, such as thermal control, structural reinforcement, and protection. Carbon-filled polymer composites are polymers that have high thermal conductivity, electrical conductivity, mechanical strength, and are lightweight. They can be used as electrodes, heat sinks, sensors, and actuators for various purposes, such as energy conversion, storage, and transmission. Epoxy composites are polymers that have high mechanical strength, stiffness, and adhesion, but low thermal stability, fire resistance, and electrical conductivity. They are widely used in the aeronautical and space fields for structural and functional applications, such as coatings, adhesives, sensors, actuators, etc. However, they need to be modified or reinforced with various additives or fillers to improve their thermal, mechanical, and electrical properties. In this regard, some studies have reported that incorporating mesoporous silica micro-filler or microencapsulated phase change materials (MPCM) into epoxy resin can enhance its thermal stability and mechanical properties. The mesoporous silica composite exhibited the highest glass transition temperature and activation energy for thermal degradation among all of the epoxy-silica nano/micro-composites. Indeed, an average activation energy value of 148.86 kJ/mol was recorded for the thermal degradation of unfilled epoxy resin. The maximum activation energy range was instead recorded for composites loaded with mesoporous microsilica. The EMC-5p50 sample showed the highest mean value of 217.6 kJ/mol. This remarkable enhancement was ascribed to the polymer invading the silica pores and forging formidable interfacial bonds.

This review paper can give a glimpse into future research directions by providing indications for improving the thermal stability of polymers and composites for aeronautics. In particular, for the analyzed materials, the remarkable future perspectives are summarized as follows:

- Multifunctional polyimides can combine different properties such as self-healing, shape memory, stimuli-responsiveness, etc. for adaptive and smart applications.
- Optimized polyimide processing methods can enable the large-scale production, precise control, and high quality of polyimide materials and devices.
- The investigation of polyimide degradation mechanisms that can reveal the effects of various environmental factors such as temperature, radiation, oxygen, etc. on the performance and durability of polyimide materials.
- The exploration of hybrid carbon polymer composites that can combine different types of carbon fillers, such as CNTs, graphene, and carbon nanofibers CNFs, to achieve synergistic effects and multifunctionality.
- The development of smart carbon polymer composites that can sense, respond, and adapt to external stimuli such as the temperature, pressure, strain, electric field, etc. For example, smart composites of CNTs and epoxy can have piezoresistive, thermoelectric, and shape memory properties that can enable self-monitoring, self-healing, and self-regulating functions. Smart composites of graphene and epoxy can have tunable electrical conductivity, thermal conductivity, and optical transparency that can enable multifunctional applications such as in sensors, actuators, and heaters.
- The optimization of the carbon polymer composite processing methods that can enable the large-scale production, precise control, and high quality of carbon polymer composite materials and devices. For example, advanced processing methods such as electrospinning, 3D printing, vacuum infusion molding, etc. can be used to fabricate carbon polymer composites with the desired shapes, sizes, structures, and properties.

## Figures and Tables

**Table 1:** Comparison of thermal properties of polymer-based composites for space applications: 5% weight loss temperature (*T*_5%_), temperature of maximum mass loss (*T_max_*), melting temperature (*T_melt_*).

Polymer Matrix	Filler	Thermal Analysis Technique	Thermal Properties	Applications	Ref.
PMMA	GNP	TG	*T*_5%_ = 219 °C (0.25 wt% GNP) *T*_5%_ = 243 °C (2 wt% GNP)	Electronics	[60]
PBI	MWCNT, GO, GO-MWCNT	TG	*T_max_* = ~471 °C (5 wt% MWCNT) *T_max_* = ~463 °C (5 wt% GO) *T_max_* = 481 °C (5 wt% GO-MWCNT)	Structures, electronics	[62]
PDMS	GNP, GNP-DNA	DSC	30 wt% GNP-DNA: endothermic peak at 148 °C (DNA degradation) 15 wt% GNP: thermal stability between −40–200 °C	Radiation monitoring	[77]
MDPE	MWCNT, GNP	DSC	*T_melt_* = 120.0 °C, *X_c_* = 53.8% (5 wt% GNP) *T_melt_* = 117.5 °C, *X_c_* = 55.7% (5 wt% MWCNT)	Radiation shielding	[70]
PU	Graphite	TG	*T_max_* = 335 °C (5 wt% graphite) *T_max_* = 355 °C (30 wt% graphite)	Radiation shielding	[71]
PVDF	MWCNT	DSC	*T_melt_* = 167.7 °C, X_c_ = 44.5% (8 wt% MWCNT)	Radiation shielding	[72]
PEEK	Wrapped MWCNT	TG	*T*_5%_ = 586 °C (9 wt% wrapped MWCNT)	Radiation shielding	[73]

*Abbreviations* PMMA: poly(methyl methacrylate); PBI: polybenzimidazole; PDMS: polydimethylsiloxane; MDPE: medium-density polyethylene; PU: polyurethane; PVDF: poly(vinylidene fluoride); PEEK: poly(ether ether ketone); GNP: graphene nanoparticles; MWCNT: multi-walled carbon nanotubes; GO: graphene oxide; DNA: deoxyribonucleic acid.

## Data Availability

Not applicable.
